# Safety and Immunogenicity of the Recombinant Mycobacterium bovis BCG Vaccine VPM1002 in HIV-Unexposed Newborn Infants in South Africa

**DOI:** 10.1128/CVI.00439-16

**Published:** 2017-02-06

**Authors:** André G. Loxton, Julia K. Knaul, Leander Grode, Andrea Gutschmidt, Christiane Meller, Bernd Eisele, Hilary Johnstone, Gian van der Spuy, Jeroen Maertzdorf, Stefan H. E. Kaufmann, Anneke C. Hesseling, Gerhard Walzl, Mark F. Cotton

**Affiliations:** a SA MRC Centre for TB Research, DST/NRF Centre of Excellence for Biomedical Tuberculosis Research, Division of Molecular Biology and Human Genetics, Faculty of Medicine and Health Sciences, Stellenbosch University, Cape Town, South Africa; b Vakzine Projekt Management, GmbH, Hanover, Germany; c HJ Clinical Trials Consultancy CC, George, South Africa; d Max Planck Institute for Infection Biology, Department of Immunology, Berlin, Germany; e Desmond Tutu TB Center, Department of Pediatrics and Child Health, Faculty of Medicine and Health Sciences, Stellenbosch University, Tygerberg, South Africa; f Fam-Cru, Department of Pediatrics and Child Health, Faculty of Medicine and Health Sciences, Stellenbosch University, Tygerberg, South Africa; Vanderbilt University Medical Center

**Keywords:** recombinant BCG, newborn, immunogenicity, VPM1002, safety, HIV-unexposed, IL-17, recombinant, vaccines, whole blood assay

## Abstract

Tuberculosis is a global threat to which infants are especially vulnerable. Effective vaccines are required to protect infants from this devastating disease. VPM1002, a novel recombinant Mycobacterium bovis bacillus Calmette-Guérin (BCG) vaccine previously shown to be safe and immunogenic in adults, was evaluated for safety in its intended target population, namely, newborn infants in a region with high prevalence of tuberculosis. A total of 48 newborns were vaccinated intradermally with VPM1002 (*n* = 36) or BCG Danish strain (*n* = 12) in a phase II open-labeled, randomized trial with a 6-month follow-up period. Clinical and laboratory measures of safety were evaluated during this time. In addition, vaccine-induced immune responses to mycobacteria were analyzed in whole-blood stimulation and proliferation assays. The safety parameters and immunogenicity were comparable in the two groups. Both vaccines induced interleukin-17 (IL-17) responses; however, VPM1002 vaccination led to an increase of CD8^+^ IL-17^+^ T cells at the week 16 and month 6 time points. The incidence of abscess formation was lower for VPM1002 than for BCG. We conclude that VPM1002 is a safe, well-tolerated, and immunogenic vaccine in newborn infants, confirming results from previous trials in adults. These results strongly support further evaluation of the safety and efficacy of this vaccination in larger studies. (This study has been registered at ClinicalTrials.gov under registration no. NCT01479972.)

## INTRODUCTION

In 1993 the World Health Organization (WHO) declared tuberculosis (TB), caused by Mycobacterium tuberculosis, a global emergency (1). Since then, the TB epidemic has escalated, despite advances in both diagnostic tests and treatment. In most adults, M. tuberculosis infection is contained by host defense mechanisms, and infection remains latent. However, among infants (<12 months of age), up to 50% of those infected develop TB disease in the absence of chemoprophylaxis (2–4). In 2015, 11% of the 10.4 million incident TB cases occurred in children (1). Although pulmonary TB is the most common form of TB in both infants and adults, disseminated TB, mainly miliary TB and TB meningitis, contributes significantly to the TB burden in children (3, 5–7). In addition to this, pulmonary TB in infants can be severe, with extensive lung involvement in up to 75% of cases (8). The burden of childhood TB is considerable and until recently was underestimated. A mathematical model to estimate the incidence of pediatric TB in 22 high-burden countries suggested that in 2010, approximately 7.6 million children in these countries became infected with M. tuberculosis, and roughly 650,000 developed TB disease (9).

Bacillus Calmette-Guérin (BCG), an attenuated strain of Mycobacterium bovis, has been used as a TB vaccine since 1921. Approximately 4 billion doses have been administered subsequently. Various strains of BCG are currently being used worldwide as part of the expanded program of immunization (EPI). In South Africa, BCG Danish SSI (10) (or BCG from the Serum Institute of India since 2016 [11]) is administered at birth as a single, intradermal injection. Its protective efficacy against TB meningitis and miliary TB disease in young children ranges between 64 and 86% (12). This is the main rationale for routine infant vaccination in high-burden countries such as South Africa, where coverage is high (98% of infants vaccinated in 2011). However, severe TB disease is still observed frequently in children. For example, in a large case series of 244 children with TB meningitis from the Western Cape, South Africa, all but 8 had received BCG (13) and, of those that survived, >75% showed neurological impairment. The protective efficacy of BCG against pulmonary TB is even less certain (1, 14–17), and yet pulmonary TB comprises >75% of the disease burden (12). Development of a safe and efficacious vaccine has been recognized as a global priority to combat the TB epidemic by the WHO Child TB Subgroup (18). Such development is challenging given the absence of known, validated biomarkers predictive of vaccine efficacy. Understanding the immunological mechanisms of M. tuberculosis infection and BCG vaccination is therefore vital.

Both M. tuberculosis and BCG are phagocytosed by host macrophages (19, 20) and retained in the cell's phagosomes. Mycobacterial antigens primarily enter the major histocompatibility complex class II (MHC-II) pathway and preferentially induce CD4^+^ T-cell responses, critical in the control of disease (21, 22). In addition, MHC-I-restricted CD8^+^ T cells are thought to play an important role in the immune response to M. tuberculosis infection (20, 23). CD8^+^ T-cell responses are, however, only weakly induced by BCG (17, 19, 20, 24–27).

The recombinant BCG vaccine VPM1002 was developed to enhance MHC-I-related immune responses and, through this, to be more effective and safer than conventional BCG (28). VPM1002 expresses listeriolysin (Hly) from Listeria monocytogenes (28, 29), which perturbs the phagosome, thereby facilitating antigen translocation into the cytoplasm and allowing efficient presentation to CD8^+^ T cells (30). In addition, the induction of apoptosis and autophagy leads to cross-priming, resulting in further stimulation of CD4^+^ and CD8^+^ T-cell responses (31–33). Importantly, VPM1002 has been shown recently to expand CD4^+^ central memory T cells (34), as well as both T helper 1 (TH1) and TH17 cells, at a more profound level than BCG (35). Similarly, CD8^+^ T cells are potently induced by VPM1002 (30). Moreover, VPM1002 has been shown to be cleared more rapidly from tissue than BCG, resulting in reduced persistence in the host (28, 32–34).

HIV-infected and immune-deficient infants are at risk of BCG-related complications (36–38) (with a risk of disseminated BCG disease [“BCGosis”] in HIV-infected infants of 992 per 100,000 children), although in high-burden TB settings BCG vaccination is routinely performed at birth before the infant's HIV status can be determined. In this setting, a vaccine safer than BCG is therefore desirable.

During development of VPM1002, inactivation of the urease C gene was performed to ensure optimal Hly activity in an acidic phagosomal environment (28). Initially, a hygromycin resistance gene was incorporated into VPM1002 to facilitate clonal selection during vaccine development (32). In 2011, however, this gene was removed successfully (39). VPM1002 is sensitive to isoniazid, rifampin, and ethambutol, all effective antimycobacterial agents (40), in the presence or absence of the hygromycin resistance gene.

VPM1002-specific human data are available from two clinical phase I trials (ClinicalTrials.gov, NCT00749034 and NCT01113281). In a phase Ia trial conducted in Germany, healthy adult Caucasian male volunteers with or without preexposure to BCG received VPM1002 (*n* = 30) or BCG (*n* = 20) and were followed up for 6 months. A single vaccination with up to 5 × 10^5^ CFU was safe and well tolerated. VPM1002 was immunogenic and induced multifunctional CD4^+^ and CD8^+^ T-cell subsets, considered relevant for protection against TB (41). Induction of multifunctional CD8^+^ T cells by VPM1002 appeared to be superior to BCG at comparable doses, but formal statistical testing was not performed. The immunogenicity of VPM1002, detected by interferon gamma (IFN-γ) release by stimulated T cells, was dose dependent (41).

In a second clinical trial, conducted in South Africa, 24 healthy male and female adults, predominantly of indigenous African descent and all with prior exposure to BCG, received VPM1002 at the same dose as in the phase Ia trial and were followed up for 6 months. A single vaccination with VPM1002 was safe, well tolerated, and immunogenic (ClinicalTrials.gov, NCT01113281) and thus confirmed results obtained in phase Ia.

The primary objective of the present study (ClinicalTrials.gov, NCT01479972) was to evaluate the safety and tolerability of VPM1002 compared to BCG in newborn infants in a setting of TB endemicity. The secondary objective was to compare immunogenicity of the two vaccines. BCG forms an integral component of the national EPI in this setting and was therefore selected over a placebo as a suitable and ethical comparator.

## RESULTS

### Clinical and laboratory measures.

**FIG 1:**
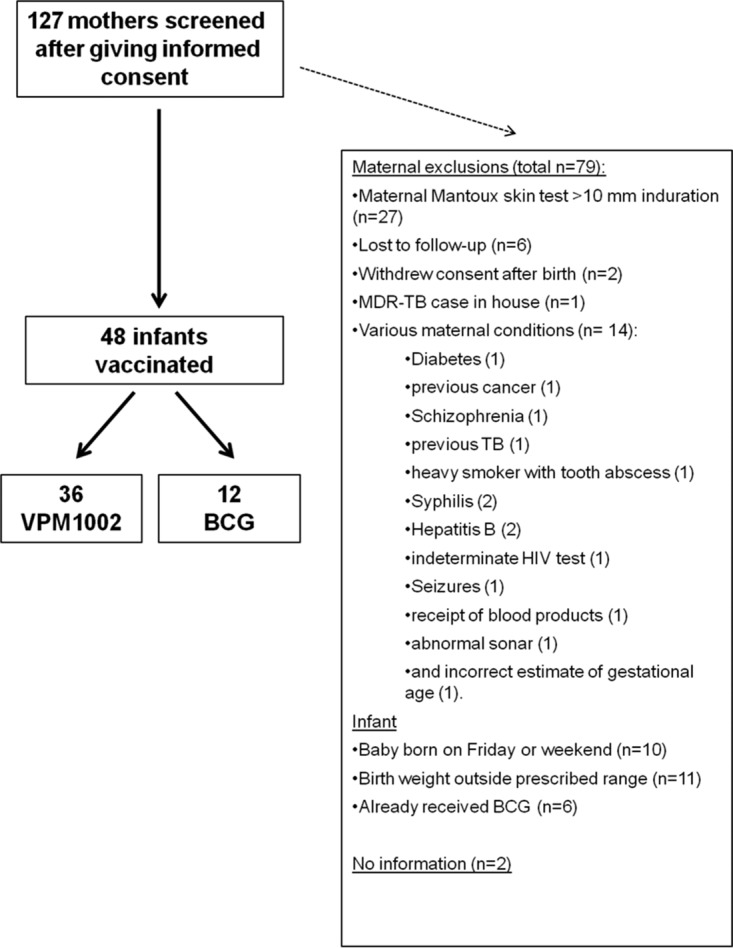
Flowchart representing consented, screened, and enrolled newborn infants of the trial. MDR-TB, multidrug-resistant TB.

Forty-eight infants were enrolled from 127 mothers screened (Fig. 1). All completed the study. Infant demographics and baseline assessments are summarized in Table 1. Other than recumbent length (*P* = 0.0392), there were no significant differences in these parameters between the two vaccination groups.

**TABLE 1:** Infant demographics and findings at screening^*a*^

Parameter	Result for infants vaccinated with:		*P*^*b*^
	BCG (*n* = 12)	VPM1002 (*n* = 36)	
Mean age in days (SD)	2.6 (1.31)	2.1 (1.27)	0.2482
No. (%) of male subjects	6 (50.0)	21 (58.3)	0.7406
Ethnic origin, no. (%) of subjects			0.4997
Caucasian	1 (8.3)	2 (5.6)	
Mixed	10 (83.3)	28 (72.2)	
Black	1 (8.3)	6 (22.2)	
Mean birth wt, kg (SD)	3.37 (0.271)	3.40 (0.270)	0.7252
Mean wt at screening, kg (SD)	3.31 (0.353)	3.35 (0.328)	0.6833
Mean Apgar score (SD)	9.8 (0.39)	9.6 (0.49)	0.1640
Mean recumbent length, cm (SD)	49.0 (1.91)	50.4 (1.98)	0.0392*
Mean mid-upper-arm circumference, cm (SD)	10.8 (0.68)	10.9 (0.70)	0.6504
Mean body temp, °C (SD)	36.5 (0.24)	36.5 (0.40)	0.8041
Mean heart rate, bpm (SD)	133 (12.9)	129 (10.1)	0.3811

a *n*, total number of subjects in each treatment group; SD, standard deviation; bpm, beats per minute.

b *P* values were obtained using ANOVA with treatment as the main effect (continuous data) or the Fisher exact test (categorical data). *, *P* < 0.05.

During the 6 months of study, 623 adverse events were reported: 155 in the BCG group and 468 events in the VPM1002 group. The incidence rates were similar in the two groups (Table 2).

**TABLE 2:** Adverse events over the study period^*a*^

Parameter	Result for infants vaccinated with:		*P*^*b*^
	BCG (*n* = 12)	VPM1002 (*n* = 36)	
Total no. of adverse events reported	155	468	
Adverse event rate, per subject-yr (95% CI)	25.82 (21.91–30.21)	25.97 (23.67–28.43)	0.9499*
No. (%) of subjects experiencing at least one adverse event	12 (100.0)	36 (100.0)	1.0000†
Intensity of adverse events, no. (%) of subjects			0.3093†
Mild	139 (89.7)	410 (87.6)	
Moderate	13 (8.4)	36 (7.7)	
Severe	3 (1.9)	22 (4.7)	
Adverse vaccine reactions^*c*^	65	189	
Vaccine-related event rate, per subject-yr (95% CI)	10.66 (8.21–13.61)	10.49 (9.05–12.09)	0.9099^*d*^
Serious adverse events (events per subject)	0 (0.0)	3^*e*^ (0.1)	1.000^*f*^

a *n*, total number of subjects in each treatment group; CI, confidence interval.

b *, Rate ratio of events in the VPM1002 group compared to the BCG group = 1.006, with a 95% CI of 0.839 to 1.206 (the *P* value was obtained from a z-test); †, determined using Pearson's chi-square test.

c That is, adverse events where the relationship to the vaccination was judged by the investigator to be “possible,” “probable,” “certain,” or “not assessable.”

d That is, the rate ratio of vaccine-related events in the VPM1002 group compared to the BCG group = 0.984, with a 95% CI of 0.741 to 1.306. The *P* value was obtained from a z-test.

e That is, breast milk jaundice, suspected meningitis, and gastroenteritis. Breast milk jaundice and suspected meningitis and were reported in the same participant, 80 days apart. The infant presented with cough and nasal obstruction for 4 days and diarrhea for a day. A short cyanotic episode was noted. The axillary temperature was 37.9°C. She had mild respiratory distress and required additional oxygen by nasal cannulae to maintain oxygenation. A chest radiograph showed perihilar streaky infiltrates. A complete blood count showed no abnormalities, and the C-reactive protein level was <0.4 mg/liter (0.0 to 10). Because of lethargy, a spinal tap was undertaken. The protein level was 0.76 g/liter (0.2 to 0.8) and glucose 3.5 mmol/liter (2.2 to 3.9). The cerebrospinal fluid showed three neutrophils/mm^3^ and 116 red cells/mm^3^. The bacterial culture was negative.

f Determined using a Fisher exact test.

Three serious adverse events (SAEs) were reported in the VPM1002 group. Two of these occurred in a single subject who was first hospitalized to receive phototherapy for presumed breast milk-related jaundice and thereafter for suspected bacterial meningitis (see Table 2, footnote *e*). The third SAE reported was an episode of gastroenteritis. All three events resolved without sequelae following appropriate therapy; none were considered related to VPM1002.

Adverse vaccine reactions occurring in more than one subject in either treatment group are reported in Table 3. Vaccination site scarring, erythema, nodule and scab formation, mass, ulceration, discoloration, and regional lymphadenopathy were reported at similar frequencies in the two vaccination groups. Abscess formation, however, was more common (*P* = 0.0321) for BCG (41.7%) than VPM1002 (11.1%). No severe adverse vaccine reactions were reported for VPM1002. One subject developed an injection site mass of moderate intensity, which resolved spontaneously. Other reactions to VPM1002 were all of mild intensity. For BCG, one subcutaneous abscess of severe intensity and another of moderate intensity were reported. Vaccination site erythema of moderate intensity was also observed. Otherwise, all remaining adverse vaccine reactions to BCG were of mild intensity.

**TABLE 3:** Adverse vaccine reactions occurring in more than one subject in either treatment group

Reaction	No. (%) of subjects		*P*^*a*^
	BCG (*n* = 12)	VPM1002 (*n* = 36)	
Scar	11 (91.7)	33 (91.7)	1.0000
Erythema	11 (91.7)	32 (88.9)	1.0000
Nodule	8 (66.7)	28 (77.8)	0.4619
Scab	6 (50.0)	16 (44.4)	0.7514
Injection site mass	4 (33.3)	10 (27.8)	0.7260
Abscess^*b*^	5 (41.7)	4 (11.1)	0.0321*
Ulcer	1 (8.3)	5 (13.9)	1.0000
Discoloration	1 (8.3)	3 (8.3)	1.0000
Regional lymphadenopathy	3 (25.0)	9 (25.0)	1.0000

a *P* values were determined using the Fisher exact test. *, *P* < 0.05.

b No subject required surgical intervention.

Table 4 summarizes the frequencies of local reactions, including maximum size or duration, noted during follow-up. The median maximum sizes of each of these reactions were similar in the two groups. However, the median duration of crusting was prolonged for BCG (78.5 days) compared to that for VPM1002 (47.0 days), but not significantly (*P* = 0.0969).

**TABLE 4:** Local injection site reactions and maximum reaction size during follow-up^*a*^

Local reaction	Result for infants vaccinated with:		*P*^*b*^
	BCG (*n* = 12)	VPM1002 (*n* = 36)	
Scarring, no. (%) of subjects	11 (91.7)	33 (91.7)	1.0000
Median size, mm (range)	5.0 (4–15)	6.0 (2–12)	0.6617
Erythema, no. (%) of subjects	11 (91.7)	32 (88.9)	1.0000
Median size, mm (range)	5.0 (1–20)	4.0 (1–12)	0.7030
Induration, no. (%) of subjects	7 (58.3)	24 (66.7)	0.7305
Median size, mm (range)	3.0 (1–12)	4.5 (1–12)	0.6133
Crusting, no. (%) of subjects	6 (50.0)	16 (44.4)	0.7514
Median duration, days (range)	78.5 (47–91)	47.0 (3–121)	0.0969
Ulcer, no. (%) of subjects	1 (8.3)	5 (13.9)	1.0000
Median size, mm (range)	2.0 (2–2)	2.0 (1–3)	1.0000
Subcutaneous abscess, no. (%) of subjects	5 (41.7)	4 (11.1)	0.0321*
Median size, mm (range)^*c*^	4.0 (2–14)	5.0 (3–5)	1.0000
Axillary lymphadenopathy, no. (%) of subjects	5 (41.7)	14 (38.9)	1.0000
Median size, mm (range)	3.0 (2–3)	3.0 (1–5)	0.6870

a *n*, total number of subjects in each treatment group.

b The *P* values for the reaction incidence and reaction size were obtained using the Fisher exact test and the Wilcoxon rank-sum test, respectively. *, *P* < 0.05.

c Two subcutaneous abscesses (one from each vaccine group) were reported as adverse events, but the size of the reaction was not measured.

Twenty grade 3 and 4 laboratory abnormalities were reported in 18 subjects (Table 5). Three clinically significant abnormalities in subjects administered VPM1002 were considered unrelated to vaccination. Two (grade 3 low hemoglobin level and grade 4 elevated total serum bilirubin) were ascribed to a urinary tract infection, whereas the third was related to breast milk jaundice (grade 4) requiring phototherapy. Breast milk jaundice occurred in 14 subjects vaccinated with VPM1002 (38.9%) and 2 vaccinated with BCG (16.7%). The difference was not statistically significant. Unconjugated bilirubin was the predominant fraction in all infants, and hemolysis was excluded through normal peripheral blood smears.

**TABLE 5:** Grade 3 or 4 laboratory-defined abnormalities after vaccination

Time point	Abnormality	No. (%) of patients^*a*^		*P*^*b*^
		BCG (*n* = 12)	VPM1002 (*n* = 36)	
Day 14	Total/unconjugated bilirubin increased	2 (16.7)	14 (38.9)	0.2887
	Hemoglobin decreased		1 (2.8)*	
	Blood potassium increased		1 (2.8)†	
Wk 6	Neutrophil count decreased		1 (2.8)	
Mo 6	Neutrophil count decreased		1 (2.8)‡	

a *n*, total number of subjects in each treatment group. *, Participant with confirmed urinary tract infection and secondary anemia; †, hemolyzed blood sample; ‡, a repeat value obtained 2 days later was normal.

b The *P* value was obtained using the Fisher exact test.

Liver and spleen ultrasonography showed no granulomas. In one subject randomized to VPM1002, an incidental renal finding of a unilateral right duplex collecting system was noted on screening ultrasound.

### Telephone follow-up after month 6.

During the 30-month poststudy follow-up, TB was clinically suspected in 11 children at a median age of 14.6 months (interquartile range [IQR], 12.7 to 24.4 months) (Table 6). Nine had contact with a TB source case (Table 6), of whom six (16.7%) had received VPM1002 and three (25.0%) BCG. One child in each vaccination group received TB treatment. Both had hilar adenopathy on chest radiograph and one had a positive Mantoux skin test (10 mm induration). After 30 months, all children demonstrated normal weight gain and were healthy.

**TABLE 6:** Participants with suspected TB during the poststudy follow-up period^*a*^

Participants with suspected TB	Result for group			*P*^*b*^
	Total (*n* = 48)	BCG (*n* = 12)	VPM1002 (*n* = 36)	
Median follow-up time from vaccination, months (IQR)	30.0 (26.9–33.0)	32.8 (28.0–33.2)	30.0 (26.8–33.0)	0.3591*
TB suspected, no. (%) of subjects	11 (22.9)	4 (33.3)^*c*^	7 (19.4)	0.4304†
Median age when TB suspected, months (IQR)	14.6 (12.7–24.4)	19.5 (13.2–29.0)	12.8 (12.2–19.3)	0.2986*
TB contacts, no. (%) of subjects^*d*^	9 (18.8)	3 (25.0)	6 (16.7)	
Hilar adenopathy, no. (%) of subjects	4 (8.3)	1 (8.3)	3 (8.3)	
TB treated, no. (%) of subjects	2 (4.2)	1 (8.3)	1 (2.8)	
IPT, no. (%) of subjects	2 (4.2)	1 (8.3)	1 (2.8)	
TB symptoms without TB contact, no. (%) of subjects^*e*^	2 (4.2)	1 (8.3)	1 (2.8)	

a *n,* total number of subjects in each treatment group; IQR, interquartile range; TB, tuberculosis; IPT, isoniazid prevention therapy.

b *, *P* value obtained using the Wilcoxon rank-sum test; †, *P* value obtained using the Fisher exact test.

c One case had two occasions of suspected TB, 6 months apart. On one of these occasions, a TB contact was identified (Mantoux test at first visit: no induration). No IPT was documented.

d Chest X ray undertaken in six contacts, two of which were normal (one from each of the VPM1002 and BCG groups).

e None investigated, all resolved.

### Immunology. (i) Vaccination with VPM1002 induces multifunctional CD4^+^ and CD8^+^ T cells.

**FIG 2:**
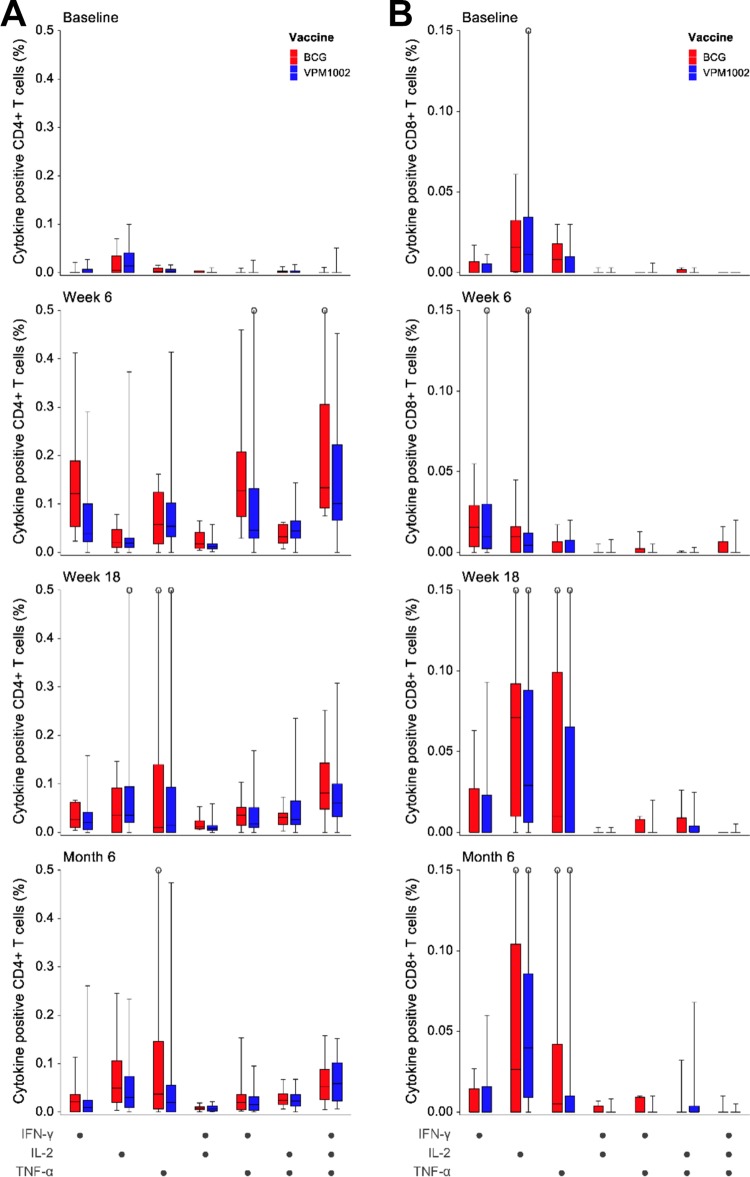
Proportions of distinct subsets of specific CD4^+^ (A) and CD8^+^ (B) T cells showing single or combined expression of IFN-γ, TNF-α, and/or IL-2 in whole blood after restimulation with PPD for 12 h. Patterns are shown for different time points before and after vaccination with BCG (*n* = 12) and VPM1002 (*n* = 36). The median proportion of each cytokine-expressing cell subset is represented by the horizontal line, the interquartile range (IQR) by the box, and the range by the whiskers. Differences in subset proportions between the vaccination groups were analyzed for each time point using a Wilcoxon rank sum test and were not significant (*P* > 0.050).

Whole-blood restimulation of samples obtained over the 6-month follow-up demonstrated CD4^+^ T cells producing multifunctional cytokine responses for both vaccines (Fig. 2A). There were no significant differences between vaccination groups after restimulation with purified protein derivative (PPD) at any time point compared to the baseline (day 14, *P* = 0.1301; week 6, *P* = 0.1122; week 18, *P* = 0.4950; month 6, *P* = 1.000). Increases in the proportions of CD8^+^ T cells after restimulation with PPD were most marked for single-cytokine-producing cells (predominantly interleukin-2 [IL-2] and tumor necrosis factor alpha [TNF-α]) at week 18 and month 6 and were comparable in the two vaccination groups at all time points (day 14, *P* = 0.3546; week 6, *P* = 0.3481; week 18, *P* = 0.0916; month 6, *P* = 0.9431) (Fig. 2B).

### (ii) Vaccine-induced IFN-γ production peaks at week 6.

**FIG 3:**
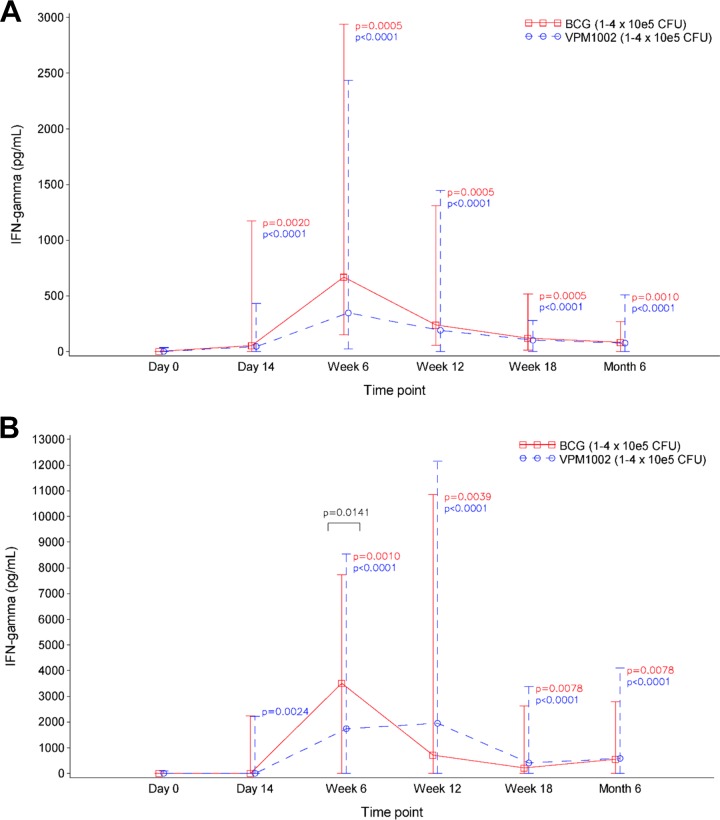
Vaccine-induced IFN-γ responses as determined by ELISA in whole-blood samples after stimulation with PPD for 7 h (A) and 7 days (B). Medians (lines) and ranges (error bars) are shown. Both BCG (*n* = 12) and VPM1002 (*n* = 36) vaccination groups showed positive IFN-γ responses to vaccination, peaking at week 6. Within each group, changes from baseline were analyzed using a Wilcoxon signed-rank test and were significant in both assays at all time points excepting BCG at day 14. The proliferative IFN-γ response in the 7 day assay was significantly greater in response to BCG than VPM1002 (*P* = 0.0141), as analyzed by a Wilcoxon rank sum test.

IFN-γ production was measured at each follow-up visit. In the 7-h assay, both BCG and VPM1002 groups had IFN-γ responses at all time points postvaccination that were significantly higher than baseline (Fig. 3A). These changes were most pronounced at week 6 (BCG, *P* = 0.0005; VPM1002, *P* < 0.0001) and were similar in each group (*P* = 0.0737).

The proliferative IFN-γ responses to 7-day PPD restimulation (Fig. 3B) reached levels five to six times higher than those from the 7-h assay. The change from baseline was significantly greater in the BCG group than in the VPM1002 group at week 6 (*P* = 0.0141).

### (iii) CD8^+^ T cells producing IL-17 are more prominent in the VPM1002-vaccinated group.

**FIG 4:**
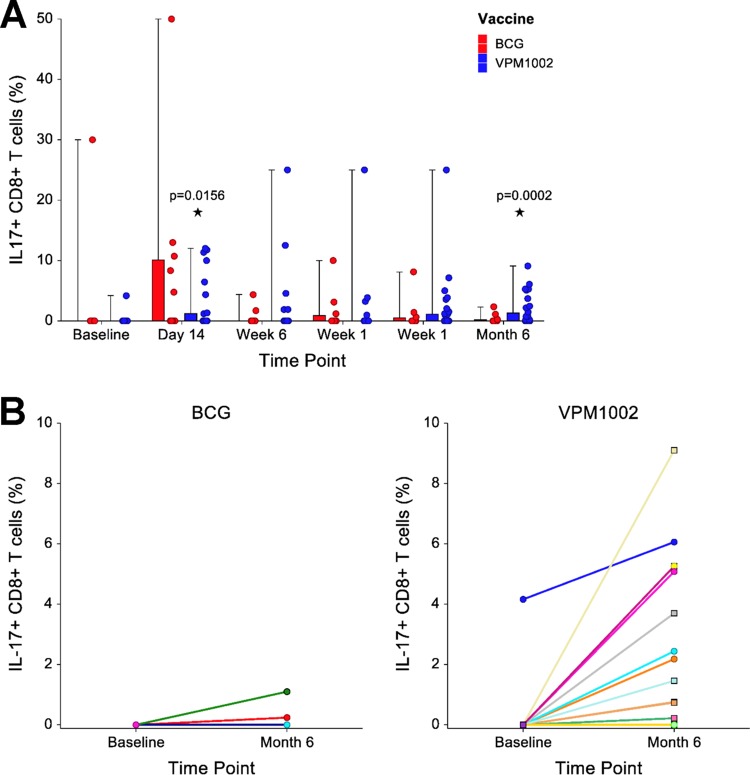
Effect of vaccination on IL-17 production by CD8^+^ T cells. (A) Proportions of CD8^+^ T cells expressing IL-17 after restimulation with BCG for 7 days. Median proportions for BCG (*n* = 12) and VPM1002 (*n* = 36) vaccination groups at each time point are expressed by the horizontal line, the interquartile range (IQR) by the box, and the range by the whiskers. Corresponding individual responses are illustrated by dots adjacent to each box-and-whisker. Changes from the baseline to each time point postvaccination were assessed using a Wilcoxon signed-rank test. These were significant in only the VPM1002 group at day 14 (*P* = 0.0156) and month 6 (*P* = 0.0002). (B) Individual longitudinal expression of IL-17 among CD8^+^ T cells from baseline to month 6 is shown for individual infants in the BCG and VPM1002 vaccination groups. Positive responses were seen in 2 (16.7%) subjects from the BCG and 13 (36.1%) from the VPM1002 vaccination groups. Median responses were similar in the two vaccination groups (*P* = 0.0836, Wilcoxon rank sum test).

Compared to the baseline, significantly increased proportions of CD8^+^ IL-17^+^ cells were seen in the VPM1002 group at day 14 (*P* = 0.0156) and month 6 (*P* = 0.0002) after restimulation with BCG in the 7-day proliferation assay but not in the BCG group (Fig. 4A). Individual subject changes in proportions of the CD8^+^ IL-17^+^ cells are given per vaccination group (Fig. 4B). The median changes from baseline to month 6 were similar in both groups (*P* = 0.0836).

### RNA expression between VPM1002 and BCG.

**FIG 5:**
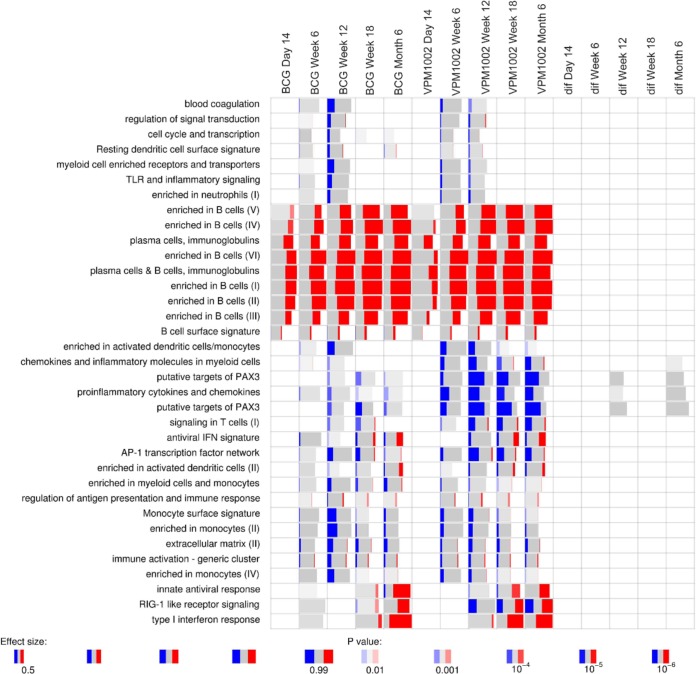
Gene set enrichment of differentially expressed genes. Enrichment was calculated for each time point relative to baseline. Red indicates the proportion of genes in a particular module which show significantly increased expression. Likewise, blue indicates significantly lower expression of genes. Modules which are gray are enriched but individual genes in that module are not significantly changed. The columns on the right indicated by “dif” show differences between the BCG and VPM1002 group at each time point.

Gene expression analysis revealed marked changes over time. Gene set enrichment analysis indicated that the most significant changes were in B-cell-related genes (Fig. 5), many of which were expressed at higher levels postvaccination. These changes were observed from 14 days postvaccination onward. Furthermore, genes related to type I IFN and innate antiviral responses showed higher expression at later time points, whereas several enriched gene modules showed decreased expression of genes over time (Fig. 5). These changes most likely reflect the maturation of the immune system and increasing proportions or activity of B cells over time.

**FIG 6:**
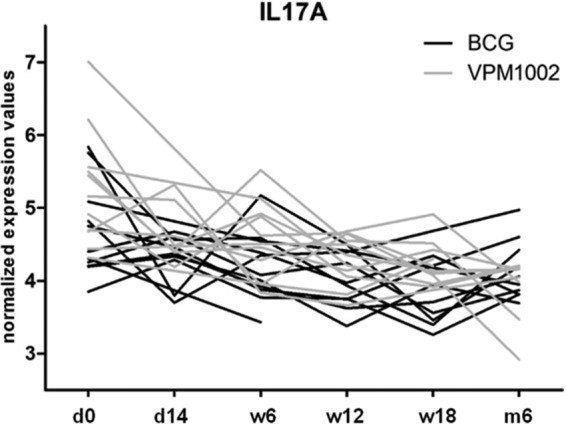
IL-17A gene expression in whole-blood samples throughout the study period from VPM1002- or BCG-vaccinated infants. d, day; w, week; m, month.

No significant differences between the VPM1002 and BCG groups were observed longitudinally (Fig. 5). The administration of VPM1002, but not of BCG, resulted in an increase in CD8^+^ IL-17^+^ cells (Fig. 4). However, no overall differences in basal IL-17A gene expression in whole blood were noted (Fig. 6). In fact, both groups showed a significant overall decrease in IL-17A gene expression level in whole blood over time, with a log_2_-fold change of −0.94 and corrected *P* value of 1.38E−06 at month 6 postvaccination.

## DISCUSSION

This phase II trial confirms the safety and immunogenicity of VPM1002 in HIV-unexposed South African newborn infants. A single VPM1002 vaccination induced polyfunctional CD4^+^ and CD8^+^ T-cell profiles comparable to BCG. Interestingly, the proportions of CD8^+^ IL-17^+^ T cells were increased at 6 months postvaccination in the VPM1002 group exclusively.

This subgroup of CD8^+^ T cells is induced in response to inflammatory stimuli, especially in an IL-6-rich environment (42). In a recent publication, this cell type was detected in patients with active TB disease, but no correlation with bacterial loads in the sputum could be found (43), and it is currently not known to what extent this cell type correlates with pathology or protection during M. tuberculosis infection.

Importantly, however, IL-17-producing CD8^+^ T cells have been shown to confer vaccine-mediated protection under various conditions (44–47). For the first time, we show here that such a cell type is also induced by vaccination with a BCG strain, namely, VPM1002. Further studies are needed to decipher the exact role of these cells in VPM1002-mediated responses.

The incidences of adverse events were similar in both groups. All subjects experienced at least one adverse event regarded as an adverse vaccine reaction (i.e., a reasonable possibility of a causal relationship with the vaccine). These were predominantly local injection site reactions, and incidence rates comparable to those reported after routine BCG vaccination were expected (10). Of note, abscess formation, an occasional reaction to BCG vaccination, was observed less frequently in VPM1002-vaccinated infants than in BCG controls. This may suggest an enhanced safety profile of VPM1002.

Throughout the study, 25 adverse events of severe intensity were reported in 21 subjects. Only one of these events (abscess formation in a subject vaccinated with BCG) was considered to be related to vaccination. Elevated bilirubin levels (grade 3 or 4) were reported on day 14 in 2 and 14 subjects from the BCG and VPM1002 groups, respectively. The Division of AIDS (DAIDS) toxicity tables used during the study did not consider bilirubin levels in breast-fed infants, which can remain elevated until 4 weeks of age. Revised toxicity tables were introduced in 2014, and had these been applied, no infant in the study would have had grade 3 or 4 hyperbilirubinemia (48).

Other abnormal laboratory results were considered unrelated to vaccination. Clinically significant findings related to urinary tract infections (two subjects) resolved upon appropriate treatment. Telephonic follow-up over 30 months suggested a high level of TB exposure. Since the study duration was only 6 months, we referred children to community healthcare facilities to manage TB exposures during telephone follow-up. The management was suboptimal, as previously noted in the same setting (49), with only four of nine TB contact-exposed children receiving either treatment or isoniazid prevention therapy (IPT).

The profiles of cytokine-producing cells were similar in the BCG and VPM1002 groups. The pattern of cytokine production (for IFN-γ, IL-2, and TNF-α) by CD4^+^ and CD8^+^ T cells was consistent with findings from phase I studies (41). Furthermore, levels of granulocyte-macrophage colony-stimulating factor (GM-CSF) production by restimulated CD4^+^ and CD8^+^ T cells from whole blood were similar in the two groups and highest by week 18 (data not shown). In whole-blood assays (WBAs), a significant increase in the proportion of CD4^+^ T cells expressing IL-17 was seen in both groups at week 18 and month 6 relative to the baseline, after stimulation with PPD and BCG antigens. The median increase in the proportion of this subgroup of cells was maximal at week 18 in both groups after stimulation with BCG (median 0.022 [*P* = 0.0059] and 0.034 [*P* < 0.0001] increases, respectively, in BCG and VPM1002 vaccination groups) (data not shown).

Of particular interest is the increased frequency of IL-17 production by CD8^+^ T cells noted exclusively in the VPM1002 group postvaccination. Transient IL-17 may contribute to protective immunity against M. tuberculosis (17, 22, 50, 51). Several studies have evaluated the importance of IL-17 (50–53), recently shown to enhance the neutrophil response to M. tuberculosis (52). Increasing evidence (54, 55) strengthens earlier findings (23, 27, 33) that CD8^+^ T cells are important for TB protection. VPM1002 was designed to stimulate CD8^+^ T cells through increased antigen loading of MHC-I through enhanced phagosome permeability mediated by Hly. Although data from this trial support this concept, the protective efficacy of this phenomenon against TB remains to be clarified.

Earlier studies argue against a role of multifunctional T cells in protection against TB (56, 57). A recent review of polyfunctional T cells described the role of such cells during M. tuberculosis infection and vaccine-induced memory responses (19). Although important in vaccine-mediated immunity, the production and levels of IFN-γ (and other cytokines) are not considered direct correlates of protection. Our data on T-cell cytokines suggest that VPM1002 is highly immunogenic and induces the favorable polyfunctional T-cell response (19).

RNA analysis revealed differential regulation of a number of genes following vaccination with VPM1002 and BCG. Genes associated with B-cell development and proliferation were the most significantly regulated and occurred 14 days postvaccination. In experimental murine TB, preferential induction of antibody responses by VPM1002 over BCG has been observed (34). Further investigation into these B-cell genes is important as such a signature may indicate protection from active TB disease in the future (58). Our finding supports data from Cliff et al. (59), who demonstrated that most changes in B-cell gene expression in active TB cases occur between week 4 and the end of successful therapy.

The pipeline of new TB vaccines comprises novel recombinant vectors, adjuvant/protein formulations and live recombinant mycobacteria (60–63). VPM1002 is an attractive candidate for the latter approach. Increased IL-17 induction by CD8^+^ T cells after vaccination with VPM1002 may contribute to protection against active TB disease.

### Conclusion.

VPM1002 was safe, immunogenic, and well tolerated in newborn infants. The conduct of larger studies, including the enrollment of HIV-exposed infants and possible cohorts across different continents, is recommended to further investigate the efficacy of VPM1002.

## MATERIALS AND METHODS

### Study population.

The present study was a single-center, open-label, randomized, controlled, single-administration phase II study conducted in Cape Town, South Africa, a region where TB is highly endemic. Participants were healthy newborn infants born to HIV-negative mothers and were followed-up for 6 months after vaccination.

### (i) Mothers.

After written consent was obtained, pregnant women aged 18 and older were screened at antenatal clinics and postdelivery wards up to 3 weeks prior to their infant's enrollment into the study. Mothers who had a history of immunodeficiency, had symptoms or signs of active or latent TB, or reported household TB contacts were excluded from participation in the trial. A maternal Mantoux tuberculin skin test (two units of Statens Serum Institut tuberculin RT23 in 0.1 ml of solution for intradermal injection) was performed at screening to exclude latent TB infection; induration of <10 mm from 48 to 72 h after administration was considered negative. Women testing positive for HIV-1 infection (fourth-generation enzyme-linked immunosorbent assay [ELISA] for HIV antibodies and p24 antigen 2 weeks prior to infant immunization), syphilis, or hepatitis B surface antigen, as well as those with current acute infectious diseases, were ineligible for participation. A maternal history or evidence of diabetes mellitus, as well as reported or suspected substance abuse, were also exclusion criteria.

### (ii) Infants.

Full-term, newborn infants were screened and vaccinated up to 8 days of age. Infants meeting the inclusion criteria were considered for participation: a birth weight of 3,000 to 4,000 g, an Apgar score of ≥9 at 5 min, the absence of eczema or infection at the intended injection site, BCG naive, and the administration of oral polio vaccine according to the EPI schedule. Adherence to the EPI schedule (apart from BCG vaccination at birth) was required for the entire study period. Participation in another clinical trial before and for 6 months after study vaccination was not permitted.

Exclusion criteria included significant congenital abnormalities, history or evidence of systemic disease or acute, chronic or intercurrent illness (including sepsis or malignancy, fever, or hypothermia). Concomitant or prior medication significantly affecting immune function (e.g., systemic corticosteroids or immunosuppressive drugs), antibiotics given prior to study vaccination, or treatment with blood products precluded participation. In addition, infants with clinically significant blood or urine laboratory abnormalities at screening were ineligible. Neonatal jaundice not considered clinically significant by the investigator was permitted.

### Study procedures. (i) Randomization and safety assessments.

After confirmation of eligibility, infants were randomized in a 3:1 ratio to receive 2.5 × 10^5^ (range, 1 × 10^5^ to 4 × 10^5^ CFU) of either VPM1002 (Δ*ureC hly*^+^ Hyg^+^ strain [manufactured at Vibalogics GmbH, Germany, according to EU-GMP and WHO TRS 979, Annex 3 {64}]) (*n* = 36) or BCG (Danish strain BCG [Statens Serum Institut, Denmark], batch 110049A) (*n* = 12). A block randomization strategy ensured balance in the vaccine treatment group sizes throughout the study recruitment period, with the first subject receiving BCG.

Subjects were observed for at least 4 h after vaccination. Safety assessments (vital signs and monitoring for local reactions and adverse events) were performed predose (vital signs only) and at 0.5, 1, and 3 h postdose (and at 6 h for the first four subjects given VPM1002).

Mothers were educated regarding the natural course of BCG ulceration and scarring. The injection site was covered with a suitable dressing for the postvaccination observational period at the clinic, and the mothers were advised to cover the infant's upper arm. Direct contact with the vaccination site was to be avoided during the first days postvaccination, and especially in the presence of ulceration, to prevent infection and/or spread to other persons, objects and the environment. Topical applications were not permitted until complete healing of the vaccination site.

The study duration (including the screening period for mothers) was approximately 7 months. Regular safety and immunogenicity assessments were conducted during this time. Liver and spleen ultrasound examinations were performed during infant screening and at week 12 postvaccination. Adverse events were coded using the Medical Dictionary for Regulatory Activities (MedDRA), version 15.0 (http://www.meddra.org). Laboratory abnormalities were graded in accordance with DAIDS criteria (DAIDS table for grading the severity of adult and pediatric adverse events, version 1.0 [65]). All grade 3 and 4 abnormalities were considered adverse events regardless of their relationship to the study vaccine.

### (ii) Sample collection.

Blood samples for immunogenicity and safety (hematology and clinical chemistry), as well as samples for urinalysis, were collected during screening and at regular follow-up intervals until month 6 postvaccination. Urine microscopy and culture were performed for samples with abnormal dipstick results. Where residual blood samples were available, a QuantiFERON-TB Gold Test was performed to detect latent TB at week 12 and month 6 postvaccination.

### Immunological analyses. (i) Whole-blood assays.

Assessment of the immunogenicity of VPM1002 and BCG was performed using a well-established assay (66). In brief, whole blood from vaccinated infants was stimulated with BCG (Danish strain) or PPD (Statens Serum Institut) for 12 h or 7 days. For the 7-day stimulation, blood was diluted 10-fold in phosphate-bufered saline (PBS) prior to stimulation. Brefeldin A (Sigma-Aldrich), a protein transport inhibitor, was added for the last 4 h of either the 12-h or the 7-day stimulation assay. Supernatant from both stimulation assays was collected prior to the addition of brefeldin A and stored until performance of cytokine analyses. After 12 h or 7 days of stimulation, the cells were treated using fluorescence-activated cell sorting (FACS) lysing solution to remove red blood cells and then stored for further analyses.

### (ii) Flow cytometry.

Multicolor flow cytometry was performed to analyze the phenotypes and functions of cells. Samples from WBAs were permeabilized, fixed, and subsequently stained with a panel of antibodies from Becton Dickinson (BD) containing anti-CD3 (Pacific Blue), anti-CD4 (V500), anti-CD8 (allophycocyanin [APC]-Cy7), anti-IFN-γ (phycoerythrin [PE]-Cy7), anti-IL-2 (peridinin chlorophyll protein [PerCP]-Cy5.5), anti-TNF (APC), anti-IL-17 (Alexa Fluor 488), and anti-GM-CSF (PE) or anti-Ki-67 (PE). Stained samples were read on a BD FACSCanto II. FlowJo, version 10 (Treestar), was used for compensation and analysis of data.

### (iii) IFN-γ ELISA.

Supernatants from WBAs were thawed, diluted, and assayed for IFN-γ production using an ELISA^PRO^ kit (Mabtech, Sweden). The assay kit has an effective range of 3.16 to 3,160 pg/ml and was performed according to the manufacturer's instructions.

Since higher concentrations of IFN-γ were expected, supernatants were diluted prior to performing the assay. Taking the respective dilution factor into account, concentrations between 31.6 and 10,000 pg/ml and between 158 and 50,000 pg/ml, respectively, could be measured in 12-h and 7-day stimulation assays.

### RNA gene expression.

After preparation of the described safety and immunogenicity assays, residual blood was divided into aliquots and stored in RNAlater solution (Sigma-Aldrich). One aliquot was used to extract RNA for gene expression analysis on Agilent whole-genome 8×60K human expression arrays and scanned at 5 μm using an Agilent scanner. Analysis of the scanned images was performed with Feature Extraction software (version 10.5.1; Agilent Technologies). The data were analyzed using the R package limma (67). Expression data were quantile normalized and log transformed. Differential expression between the two groups of infants at various time points postvaccination was calculated based on log_2_-fold changes in an average expression. Genes with corrected *P* values of <0.01 were considered significant after Benjamini-Hochberg correction for multiple testing. Functional enrichment of differentially expressed genes was analyzed based on human blood transcriptional modules using the tmod package (68).

### Poststudy follow-up.

After completion of study-related procedures, telephonic follow-up continued for 30 months. Mothers were interviewed to obtain information regarding TB symptoms and exposure, as well as the general health of the infant. The duration of follow-up until 3 years of age was intended to cover the period for which BCG is known to effectively protect children from severe forms of mycobacterial disease (12).

### Statistical analysis.

No formal sample size calculation was performed. Both per-protocol and intent-to-treat analyses were conducted. Demographic and baseline data were presented descriptively. The Fisher exact test and analysis of variance (ANOVA) were used to test for statistically significant differences between the vaccination groups for categorical and continuous data, respectively. For the secondary immunological endpoints, descriptive statistics of the absolute values and change from baseline were presented for each time point. Change from baseline within each treatment group was analyzed for each time point using a Wilcoxon signed-rank test. Differences in the mean change from baseline between the treatment groups were analyzed using a Wilcoxon rank sum test. Point estimates of the between-group differences were calculated using the Hodges-Lehman estimate. All statistical analyses were interpreted as statistically significant for *P* values of <0.050. Statistical analysis was performed using SAS (version 9.2; SAS Institute, Inc., Cary, NC).

### Ethical and regulatory approval of the trial.

The Medicines Control Council of South Africa and the Research Ethics Committee of Stellenbosch University approved the protocol. The trial was conducted according to the Declaration of Helsinki (69) and the ICH Guideline for Good Clinical Practice (July 2002). An independent data safety monitoring board reviewed study conduct and outcomes.

### Accession number(s).

Gene expression data are deposited in the Gene Expression Omnibus (GEO) database (GSE86627).
